# Effect of Codon Optimisation on the Production of Recombinant Fish Growth Hormone in *Pichia pastoris*

**DOI:** 10.1155/2014/514835

**Published:** 2014-07-22

**Authors:** Hussin A. Rothan, Teh Ser Huy, Zulqarnain Mohamed

**Affiliations:** ^1^Institute of Research Management & Monitoring, Deputy Vice Chancellor (Research & Innovation) Building, University of Malaya, 50603 Kuala Lumpur, Malaysia; ^2^Genetics and Molecular Biology Unit, Institute of Biological Sciences, Faculty of Science, University of Malaya, 50603 Kuala Lumpur, Malaysia

## Abstract

This study was established to test the hypothesis of whether the codon optimization of fish growth hormone gene (*FGH*) based on* P. pastoris* preferred codon will improve the quantity of secreted rFGH in culture supernatant that can directly be used as fish feed supplements. The optimized *FGH *coding sequence (*oFGH*) and native sequence (*nFGH*) of giant grouper fish (*Epinephelus lanceolatus*) were cloned into* P. pastoris* expression vector (pPICZ*α*A) downstream of* alcohol oxidase* gene (*AOX1*) for efficient induction of extracellular rFGH by adding 1% of absolute methanol. The results showed that recombinant* P. pastoris* was able to produce 2.80 ± 0.27 mg of oFGH compared to 1.75 ± 0.25 of nFGH in one litre of culture supernatant. The total body weight of tiger grouper fingerlings fed with oFGH increased significantly at third (*P* < 0.05) and fourth weeks (*P* < 0.01) of four-week experiment period compared to those fed with nFGH. Both oFGH and nFGH significantly enhanced the final biomass and fish survival percentage. In conclusion, codon optimization of *FGH* fragment was useful to increase rFGH quantity in the culture supernatant of* P. pastoris* that can be directly used as fish feed supplements. Further studies are still required for large scale production of rFGH and practical application in aquaculture production.

## 1. Introduction

Fish growth hormone (FGH), a protein hormone with molecular weight of 22 kDa, is produced by somatotroph cells in the anterior pituitary gland of fish. It regulates growth and development in fish [1, 2]. Using recombinant FGH as a supplement in the feed, fish growth rate was increased without accumulation of FGH in the fish body [3, 4]. In aquaculture applications, fish growth rate has been increased after using recombinant FGH expressed in* E. coli *[5]. However, the low capacity of posttranslation process in* E. coli* resulted in less active recombinant proteins and formation of insoluble inclusion bodies [6]. Insoluble inclusion bodies require more steps in protein purification process and a complicated procedure for protein refolding to recover its biological function. It has also been known that* E. coli* is a prokaryote and its intrinsic characteristics differ from those of eukaryotes, such as protein processing, protein folding, and posttranslational modifications [7]. Fish growth hormone has been expressed in the yeast* Saccharomyces cerevisiae* [8–10] and the yeast* Pichia pastoris* [11]. The yeast* P. pastoris* expression system offers advantages over* S. cerevisiae* in its high productivity, efficient secreted expression, and stable genetics, so it has been an attractive candidate for production of foreign proteins [12]. Intracellular expression of FGH in* P. pastoris* that is used as feed supplement showed significant increase in growth rate on tilapia [13], but the expression of recombinant FGH was low (1-2% of the total cellular proteins). Interestingly,* P. pastoris* has a higher secretory capacity and lower expression level of endogenous proteins than other yeasts. Recombinant proteins comprise the majority of the total secreted proteins in the medium [14]. Of note, fish growth hormone cDNA was used in most of previous studies to produce recombinant FGH in different expression systems [5, 8–11]. In our study, we constructed a synthetic* FGH* gene with preferred codons of* P. pastoris* in order to increase the expression level of recombinant FGH. Additionally, producing extracellular FGH can omit a purification process and reduce the cost of production in fish farming.

## 2. Materials and Methods

### 2.1. Culture Media

For cloning purposes,* E. coli* strain TOP10 was cultured in low salt LB medium (LSLB) and LSLB-agar with Zeocin (salt concentration <90 mM, pH 7.5 for Zeocin to be active). The solid medium (LSLB-agar) contains 1% peptone, 0.05% NaCl, 0.5% yeast extract, and 1.5% agar with 25 *μ*g/mL Zeocin and the liquid medium was YPD broth, containing 2% peptone, 1% yeast extract, 2% dextrose, and 100 mg/L Zeocin.* P. pastoris* was cultured on YPDS-agar containing 2% agar and 18% sorbitol. For expression purposes, the buffered complex media, BMMY and BMGY, containing 2% peptone, 1% yeast extract, 4 × 10^−5^% biotin, 1.34% yeast nitrogen base, 0.1 M potassium phosphate buffer (pH 6.0), and 1% glycerol (for BMGY growth medium) or 1% methanol (for BMMY induction medium) were used.

### 2.2. DNA Preparation of* FGH* Fragments

Total RNA was isolated from the pituitary glands of giant grouper fish (*Epinephelus lanceolatus*) using RNA extraction kit (Invitogen, The Netherlands). The native* FGH* (*nFGH*) gene was reverse-transcripted and amplified using reverse transcriptase kit (Invitrogen, The Netherlands). The* nFGH* gene fragment was cloned into pGEM-T cloning vector (Promega, USA) and transformed into* E. coli* strain JM109. DNA sequencing was performed and the sequence was compared with NCBI database (http://www.ncbi.nlm.nih.gov/) for verification.

The codon-optimized FGH (oFGH) sequence was synthesised according to the* P. pastoris* preferred codons by Invitrogen (http://www.invitrogen.com/genesynthesis). Depending on synthesiser information, the following sequence regions were avoided or amended: (i) very high (>80%) or very low (<30%) GC content, (ii) the* cis*-acting sequence motifs, such as internal TATA-boxes, chi-sites, and ribosomal entry sites, and (iii) AT-rich or GC-rich sequence that stretches RNA instability motifs repeat sequences and RNA secondary structures splice donor and acceptor sites in higher eukaryotes. 

### 2.3. Construction of Expression Vectors,* pPICZ*α*A-nFGH* and* pPICZ*α*A-oFGH*

Construction of recombinant* PICZ*α*A-nFGH* and* PICZ*α*A-oFGH* was carried out as descried previously [15]. In brief, the pGEM-T cloning vectors containing* nFGH* or* oFGH* gene fragments were digested with* Eco*RI and* Not*I restriction enzymes and the fragments with molecular weight of about 600 bp were purified using QIAquick Gel Extraction kit (Qiagen, USA). The purified fragments were cloned separately into a* Eco*RI- and* Not*I-digested pPICZ*α*A vector. The recombinant plasmids, pPICZ*α*A-*nFGH* and pPICZ*α*A-*oFGH,* were transformed into* E. coli* strain Top10 of for propagation purpose. These recombinant plasmids were then isolated, sequenced, and linearized with* Sac*I restriction enzyme. Subsequently, linearized pPICZ*α*A-*nFGH* and pPICZ*α*A-*oFGH* were introduced into* P. pastoris*, wild-type X-33 strain using EasySelect* Pichia* Expression kit (Invitrogen, The Netherlands).

## 3. Expression in* P. pastoris*

Production of recombinant FGH in* P. pastoris* was carried out as described previously [15]. In brief, a single colony of recombinant X-33 harbouring nFGH and oFGH was inoculated in BMGY medium, respectively, and grown at 30°C until OD_600_ was 2–6. Cell pellet was collected by centrifugation and resuspended in BMMY media (or BGMY medium for control culture) at OD_600_ = 1. Incubation was continued at 30°C with shaking at 220 rpm. Methanol was added into BMMY medium to a final concentration of 0.5% for 12-hour intervals while glycerol was added to the BMGY medium as a substitute for methanol. Culture supernatants were harvested at 24, 36, 48, 60, and 72 hours after induction and analysed by native SDS-PAGE and SDS-PAGE under denaturing and nondenaturing conditions as described previously [15]. The recombinant protein concentrations in culture supernatant were determined using fish growth hormone, FGH ELISA kit (Cat. no. E0044f, China, http://www.eiaab.com/).

### 3.1. Bioactivity Test

Tiger grouper fingerlings were divided randomly into four groups, 20 per group (control 1 was fed with common fish feed; control 2 was fed with common feed mixed with 5% culture supernatant from wild-type* P. pastoris*; treatment 1 was fed with common feed mixed with 5% culture supernatant from recombinant* P. pastoris* producing nFGH; treatment 2 was fed with common feed mixed with 5% culture supernatant from recombinant* P. pastoris* producing oFGH). The average body weight of fingerlings was approximately 10.5 g and the average body length was 8.5 cm. Tanks and air stones were cleaned, disinfected, and refilled with new treated fresh water. New treated fresh water was replaced every 7 days for 31 days of experiment period. Feed distribution was done three times per day: 9:00 a.m., 1:00 p.m., and 6:00 p.m. Sampling was carried out every seven days. The supernatant of recombinant yeast culture was mixed with fish feed by 5% of total feed weight.

## 4. Results and Discussion

**Figure 1:**
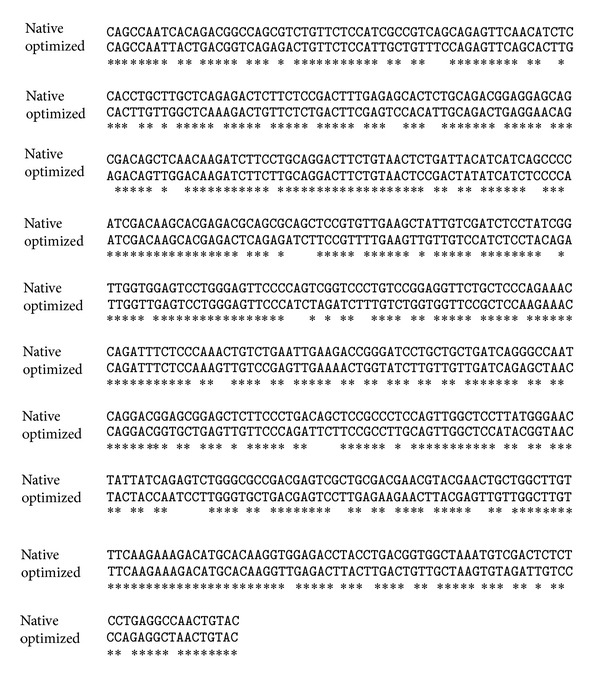
Sequences aligmnent of native fish growth hormone (nFGH) and optimized sequence based on* P. pastoris* preferred codons.

Purification and administration methods of recombinant fish growth hormone (FGH) are the main concerns of impracticable use in mass-scale aquaculture. Therefore, we conducted this study to test the hypothesis whether the optimized DNA sequence of FGH will significantly enhance its expression level in* P. pastoris* in order to be used as fish feed supplement. The entire FGH coding sequence was constructed based on* P. pastoris* preferred codons (oFGH) while the native* FGH* (*nFGH*) gene was obtained by reverse transcription of total RNA that was extracted from fish pituitary gland (Figure 1). The pPICZ*α*A plasmid contains* Saccharomyces cerevisiae* *α*-factor secretion signal peptide for extracellular protein secretion. Both DNA fragments were cloned into* P. pastoris* expression vector (pPICZ*α*A) downstream *α*-factor secretion signal peptide and the promoter of* alcohol oxidase* gene (*AOX1*) for efficient induction of extracellular FGH production by adding 1% of absolute methanol.

**Figure 2:**
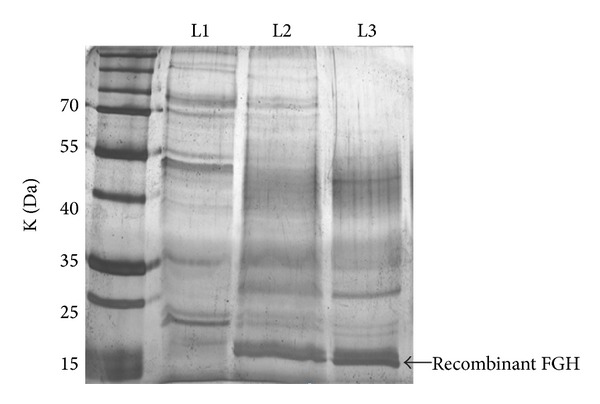
Production of native FGH (nFGH) and optimized FGH (oFGH) in* P. pastoris*. The expression vectors that contain oFGH and nFGH were transformed separately into* Pichia pastoris* strain X-33 using EasySelect* Pichia* Expression kit. A pilot experiment was carried out to identify the ability of* P. pastoris* to produce recombinant FGH as an extracellular protein. The SDS-PAGE result showed that the recombinant nFGH and oFGH were produced by* P. pastoris* at expected size of 22 kDa. L1 = control; L2 = nFGH; L3 = oFGH.

**Figure 3:**
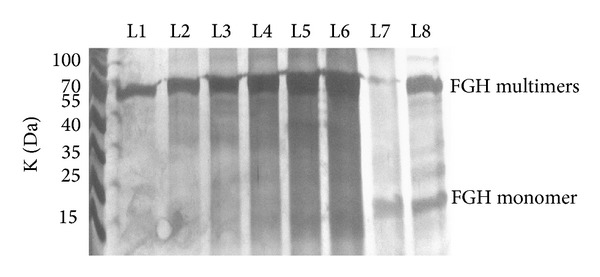
SDS-PAGE analysis of oFGH under denaturing and nondenaturing conditions. Recombinant* P. pastoris* was grown in BGMY media for 48 hrs and then transferred to induction media (BMMY) with continuous induction with 1% absolute methanol for every 12 hrs. The supernatant samples were collected at 12 (L1), 24 (L2), 36 (L3), 48 (L4), 60 (L5), and 72 hrs (L6). The high molecular weight of oFGH multimers (approximately 70 kDa) was observed when the samples were applied for SDS-PAGE without denaturing conditions (heating and reducing by beta-mercaptoethanol (L1–L6)). However, after applying the denaturing conditions only monomer molecules of oFGH were observed at 22 kDa (L7), whilst reducing the samples with beta-mercaptoethanol without heating led to detection of both oFGH monomer (22 kDa) and multimers (70 kDa) (L8).

**Figure 4:**
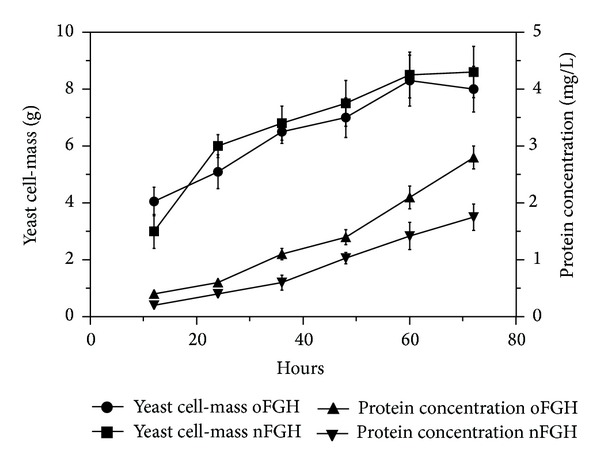
Production of recombinant oFGH and nFGH in* Pichia pastoris*. The yeast was cultured for 72 hrs with induction each 12 hrs with 1% absolute methanol. The supernatant samples were collected at 12, 24, 36, 48, 60, and 72 hrs and recombinant protein concentration was measured by FGH ELISA. The results showed that the production of oFGH was significantly higher (*P* < 0.05) than nFGH in one litre culture whereas the total yeast cell-mass was approximately similar (two-tailed paired* t*-test).

The results showed that the expression of nFGH and oFGH were detected by SDS-PAGE under denaturing conditions at the expected size of 22 kDa (Figure 2). Further analysis by SDS-PAGE under denaturing and nondenaturing conditions showed that both FGH forms (nFGH and oFGH) were produced by* P. pastoris* as monomers and multimers (Figure 3). After induction of recombinant yeast with methanol, the yeast was grown for 72 hrs, and then the production of FGH was quantified by FGH ELISA assay using standard FGH as a reference. The results showed that recombinant* P. pastoris* was able to produce 2.80 ± 0.27 mg of oFGH compared to 1.75 ± 0.25 mg of nFGH in one litre of culture supernatant (Figure 4).

**Figure 5:**
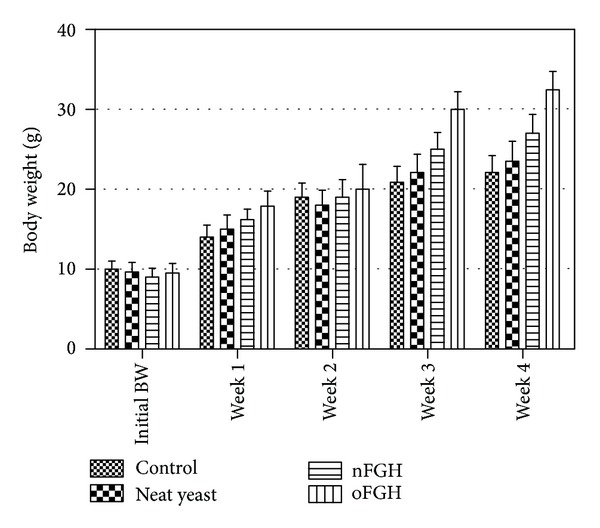
The body weight mean of fish fed with culture supernatant from recombinant* P. pastoris* producing FGH. The results showed that tiger grouper fingerlings group fed with oFGH have significantly higher body weight compared to those fed with nFGH at week 3 (*P* < 0.05) and week 4 (*P* < 0.01). Both groups fed with nFGH or oFGH showed significant higher body weight compared to neat yeast and control groups at weeks 3 and 4 (*P* < 0.05, nFGH; and *P* < 0.01, oFGH) (two-way ANOVA with bonferroni posttest).

In this study,* P. pastoris* was more efficient in producing oFGH compared to nFGH as secreted protein. It has been shown that codon optimization is important to increase translation rate through the direct use of host cell tRNA pool [16, 17] which ultimately could lead to increasing the recombinant protein quantity [18–20]. Therefore, significant increase was observed in the body weight of tiger grouper fingerlings fed with oFGH at third and fourth weeks of the experiment period compared to those fed with nFGH (Figure 5). This increase in the body weight probably was due to the higher concentration of oFGH compared to nFGH in the culture supernatant (2.80 ± 0.27 mg versus 1.75 ± 0.25 mg, resp.). The main outcome of the high content of oFGH in the culture supernatant is enhancing the bioavailability of FGH that essentially stimulates fish appetite as well as feed conversion rate [21, 22].

**Figure 6:**
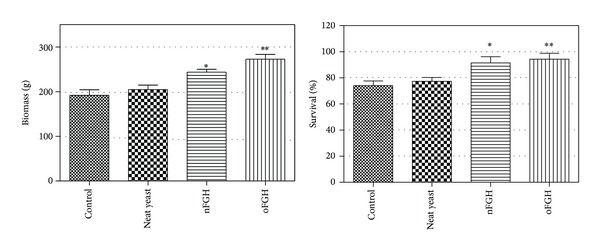
The effect of recombinant FGHs on total biomass and fish survival percentage. The results showed that total biomass (total group weight) of tiger grouper fingerlings fed with recombinant oFGH was significantly (*P* < 0.01) higher than those fed with recombinant nFGH, and both FGH groups were significantly higher than those in neat yeast group and control group (*P* < 0.05, nFGH; *P* < 0.01, oFGH). The survival percentage of fingerlings in nFGH- and oFGH-feeding groups was higher (*P* < 0.05) than those in neat yeast and control groups. However, there was no significant difference between nFGH and oFGH groups (one-way ANOVA test).

It has been known that the administration of FGH enhances many aspects of immune functions including nonspecific defences and cytotoxic, phagocytic, haemolytic, and lysozyme activities and activates immunoglobulin production as a specific defence [23]. Interestingly, it has been found that the culture supernatant containing truncated fish growth hormone has a stronger effect over growth and immune system than cells lysate containing intact growth hormone expressed in* P. pastoris* [24]. These observations are concomitant with our finding that the final biomass and fish survival percentage were significantly enhanced after feeding with culture supernatant from recombinant* P. pastoris* producing FGHs (Figure 6). In conclusion, the results of this study showed practical and cost-effective administration method of using FGH in mass-scale aquaculture through increasing the expression level of rFGH in yeast supernatant. Further studies are required for practical application in aquaculture farms.

## Abbreviations

FGH: Fish growth hormone
nFGH: Native fish growth hormone protein without codon optimization
oFGH: Fish growth hormone protein after codon optimization
rFGH: Recombinant fish growth hormone
AOX1: Alcohol oxidase gene.

## Highlights

Fish growth hormone (FGH) has been used as a supplement in fish feed to influence growth and development of farmed fish. FGH can be produced* in vitro *using molecular biology techniques, and, while many successes have been reported, several problems pertaining to the quantity and quality of the recombinant FGH still exist. In this study, a significant improvement in yield and function of recombinant FGH was achieved in* Pichia pastoris *using a recombinant gene that had been codon-optimized and expressed through the secretory pathway. This improvement should be useful for consideration in major-scale production of recombinant FGH intended for fish farming.
